# Renal transplantation outcomes in obese patients: a French cohort-based study

**DOI:** 10.1186/s12882-021-02278-1

**Published:** 2021-03-05

**Authors:** Y. Foucher, M. Lorent, L. Albano, S. Roux, V. Pernin, M. Le Quintrec, C. Legendre, F. Buron, E. Morelon, S. Girerd, M. Ladrière, D. Glotz, C. Lefaucher, C. Kerleau, J. Dantal, J. Branchereau, M. Giral, for the DIVAT consortium, Gilles Blancho, Julien Branchereau, Diego Cantarovich, Agnès Chapelet, Jacques Dantal, Clément Deltombe, Lucile Figueres, Claire Garandeau, Magali Giral, Caroline Gourraud-Vercel, Maryvonne Hourmant, Georges Karam, Clarisse Kerleau, Aurélie Meurette, Simon Ville, Christine Kandell, Anne Moreau, Karine Renaudin, Anne Cesbron, Florent Delbos, Alexandre Walencik, Anne Devis, Lucile Amrouche, Dany Anglicheau, Olivier Aubert, Lynda Bererhi, Christophe Legendre, Alexandre Loupy, Frank Martinez, Rébecca Sberro-Soussan, Anne Scemla, Claire Tinel, Julien Zuber, Pascal Eschwege, Luc Frimat, Sophie Girerd, Jacques Hubert, Marc Ladriere, Emmanuelle Laurain, Louis Leblanc, Pierre Lecoanet, Jean-Louis Lemelle, Lionel Badet, Maria Brunet, Fanny Buron, Rémi Cahen, Sameh Daoud, Coralie Fournie, Arnaud Grégoire, Alice Koenig, Charlène Lévi, Emmanuel Morelon, Claire Pouteil-Noble, Thomas Rimmelé, Olivier Thaunat, Sylvie Delmas, Valérie Garrigue, Moglie Le Quintrec, Vincent Pernin, Jean-Emmanuel Serre

**Affiliations:** 1 INSERM UMR 1246 - SPHERE, Nantes University, Tours University, Nantes, France; 2 Centre Hospitalier Universitaire de Nantes, Nantes, France; 3 CRTI UMR 1064, Inserm, Université de Nantes; ITUN, CHU Nantes; RTRS Centaur, Nantes, France; 4 Department of Nephrology and Renal Transplantation, Hospital Pasteur, Nice, France; 5 Nephrology, Dialysis and Transplantation Department, Lapeyronie University Hospital, Montpellier, France; 6 Kidney Transplant Center, Necker University Hospital, APHP, RTRS « Centaure », Paris Descartes and Sorbonne Paris Cité Universities, Paris, France; 7 Nephrology, Transplantation and Clinical Immunology Department, RTRS « Centaure », Edouard Herriot University Hospital, Hospices Civils, Lyon, France; 8 Renal Transplantation Department, Brabois University Hospital, Nancy, France; 9 Department of Nephrology and Renal Transplantation, CHU Paris-GH St-Louis Lariboisière F. Widal, Paris, France; 10 Centre d’Investigation Clinique en Biothérapie, Centre Hospitalier Universitaire de Nantes, Nantes, France

**Keywords:** Obesity, Kidney transplantation, Cohort study, Post-transplant outcomes

## Abstract

**Background:**

Whilst there are a number of publications comparing the relationship between body mass index (BMI) of kidney transplant recipients and graft/patient survival, no study has assessed this for a French patient cohort.

**Methods:**

In this study, cause-specific Cox models were used to study patient and graft survival and several other time-to-event measures. Logistic regressions were performed to study surgical complications at 30 days post-transplantation as well as delayed graft function.

**Results:**

Among the 4691 included patients, 747 patients were considered obese with a BMI level greater than 30 kg/m^2^. We observed a higher mortality for obese recipients (HR = 1.37, *p* = 0.0086) and higher risks of serious bacterial infections (HR = 1.24, *p* = 0.0006) and cardiac complications (HR = 1.45, *p* < 0.0001). We observed a trend towards death censored graft survival (HR = 1.22, *p* = 0.0666) and no significant increased risk of early surgical complications.

**Conclusions:**

We showed that obesity increased the risk of death and serious bacterial infections and cardiac complications in obese French kidney transplant recipients. Further epidemiologic studies aiming to compare obese recipients versus obese candidates remaining on dialysis are needed to improve the guidelines for obese patient transplant allocation.

**Supplementary Information:**

The online version contains supplementary material available at 10.1186/s12882-021-02278-1.

## Background

According to the World Health Organization report [1], obesity has tripled worldwide since 1975 and 650 million adults were classified as obese in 2016. Obesity is defined by a body mass index (BMI) greater than or equal to 30 kg/m^2^. Obesity is associated with increased rates of cardiovascular diseases, diabetes, musculoskeletal disorders and cancer [2]. Obesity also increases the risk for chronic kidney disease and its progression to End-Stage Renal Disease (ESRD) [3]. In the North-American patients, obesity rates have increased by 33% from 1995 to 2002 [4]. In France, the prevalence of obesity is around 21% in patients undergoing hemodialysis, whilst an increase in survival for ESRD patients with a high-level BMI have been reported [5, 6].

In ERSD patients, transplantation is recognized as the best long-term treatment compared to dialysis in terms of both the quality and the quantity of life [7, 8]. Unfortunately, obesity may be an obstacle to transplantation access, with the waiting time on dialysis of obese patients depending on center/country practices and guidelines [9–11]. In France, obese patients have a transplantation likelihood lower than non-obese ones [12]. Several reasons can explain this situation, especially the increased risk of complications after transplantation such as wound healing, delayed graft function, hospital readmissions, or new-onset diabetes [13, 14]. In contrast, the literature remains controversial concerning graft and patient outcomes. Several recent meta-analyses illustrate this debate [15–19].

Nicoletto et al. [15] reported that the only consequence of obesity was a higher incidence of Delayed Graft Function (DGF). Lafranca et al. [16] showed no significant difference between obese and non-obese patients in terms of patient and graft survival. In contrast, the authors observed a protective relationship between high-level BMI and 3-year mortality. Hill et al. [17] reported no significant correlation between obesity and patient survival, a weak relationship with death-censored graft survival, and a correlation with DGF. Sood et al. [18] reported that obese patients had an increased risk of DGF, acute rejection, death and death-censored graft failure.

The literature based on studies from European countries is weak or underrepresented, and most studies have assessed only North-American patients [15–18]. More specifically, no study based on a large cohort of French patients has been performed, with the largest including only 250 patients [19]. In this context, the aim of our study was to analyze the relationships between obesity and post-transplant outcomes by using a large and multicentric cohort of French kidney transplanted recipients.

## Methods

### Studied population

Data were extracted from the French prospective DIVAT (*Données Informatisées et VAlidées en Transplantation*) cohort (www.divat.fr, French Research Ministry: RC12_0452, last agreement No 13334, No CNIL for the cohort: 891735) composed of kidney transplant recipients followed in Nantes, Paris (Necker and Saint-Louis), Montpellier, Nancy, Lyon, and Nice University Hospitals. This represents around one third of the kidney transplantations performed in France. The study was performed in accordance with the Declaration of Helsinki and we obtained the agreement of the DIVAT Scientific Council. The quality of the DIVAT data bank is validated by a center audit. The participants gave written informed consent. The included patients were older than 18 years with a BMI-level higher than 18.5 kg/m^2^ at the time of their transplantation. Only first kidney transplants from deceased donors were considered. Multiple organ transplant recipients were not included. The study was limited to transplantations performed from January 2005 to December 2016 to respect current practices. Patients were considered in the obese group if their BMI at transplantation was higher than 30 kg/m^2^.

### Available data at transplantation

Donor variables extracted from the database were age, sex, last serum creatininemia, donor cause of death and type (living or deceased including heart or non-heart beating donors), and expanded donor criteria (ECD).^20^ Recipient characteristics at baseline were age, sex, blood group, initial recurrent causal nephropathy following transplantation (the following were considered as possibly recurrent: glomerulosclerosis, serious nephrotic syndrome with focal sclerosis, IgA nephropathy (Berger’s disease), dense deposit disease, glomerulonephritis, Wegener’s granulomatosis, Lupus erythematosus, Henoch-Schoenlein purpura, Goodpasture’s syndrome, systemic sclerosis (scleroderma), haemolytic uraemic syndrome, multi-system disease), renal disease, comorbidities prior to transplantation (diabetes, hypertension, dyslipidemia, neoplasia, cardiovascular history (cardiopathy and cardiomyopathy, cardiac insufficiency, coronaropathy, cardiac rhythm disorder, cardiac valvopathy, cardiac valvular prothesis, cardiac conduction disorder, pacemaker, cardiogenic shock or collapses, pulmonary hypertension, cerebrovascular accident (stroke or bleeding), peripheral arteriopathy, or venous thrombo-embolism), duration on waiting list before transplantation, type of dialysis before transplantation (peritoneal, hemodialysis or pre-emptive) and anti- Human Leucocyte Antigen (HLA) class I or anti-class II immunization before transplantation. Transplantation parameters were cold ischemia time, number of HLA (A + B + DR) incompatibilities and induction therapy. For donor and recipients, Epstein-Barr Virus (EBV) and Cytomegalovirus (CMV) serology were also extracted.

### Post-transplantation outcomes

The long-term outcomes were the patient and graft survival (defined by the time between the transplantation and the first event between return to dialysis, pre-emptive re-transplantation or death), graft survival (death with a functioning graft were right-censored) and patient survival (return to dialysis or pre-emptive re-transplantation were right-censored). The mid-term outcomes were the time to the first biopsy-proven acute rejection episode, cardiac complication (cardiopathy and cardiomyopathy, cardiac insufficiency, coronaropathy, cardiac rhythm disorder, cardiac valvopathy, cardiac valvular prothesis, cardiac conduction disorder, pacemaker, cardiogenic shock or collapses, pulmonary hypertension), serious bacterial infection (endocarditis, mediastinitis, myocarditis, valve prosthesis infection, pericarditis, dermohypodermatitis, colitis, biliary tract infection, pancreatitis, peritonitis, salpingitis, hepatitis, liver abscess, meningoencephalitis, meningoradiculitis, brain abscess, pulmonary abscess, pleural infection, pneumonia, osteoarthritis, or pyelonephritis) and cancer (solid, skin and Post-Transplantation Lymphoproliferative Disorders - PTLD). The short-term outcomes were the surgical or renal vascular complications occurring within the first month post-transplantation. Two sub-group analyses were performed to study DGF occurrence (defined by the need for dialysis in the first week post-transplantation) and the time to New Onset Diabetes After Transplantation (NODAT). The analysis of DGF excluded the pre-emptive patients and those on peritoneal dialysis (because one cannot measure a DGF in this subpopulation), whereas the analysis of the time to NODAT excluded the diabetic patients prior to transplantation.

### Statistical analyses

The characteristics between the two groups of interest (obese and non-obese recipients) were compared using Chi-square tests for categorical variables and Student t-tests for continuous variables. Survival curves were obtained by using the Kaplan-Meier estimator. To further compare the outcomes and to consider possible confounders, multivariate logistic regressions were used for binary outcomes and cause-specific Cox models for times-to-event. Variables significantly associated with both the outcome and the obesity status in univariate regressions were retained (*p* < 0.20) in the multivariable models. We did not consider the treatments as covariates since they constituted consequences of the obesity status. The consideration of such covariates on the pathway would have been associated with over-adjusted results. The log-linearity assumption was automatically checked: rejection of this assumption occurred when the Bayesian Information Criterion decreased using natural spline transformation compared to the inclusion of the covariate in its natural scale. In cases of violation, variables were categorized. Hazard proportionality was checked by plotting log-minus-log survival curves according to the two groups of interest and studying the Schoenfeld residuals. The collinearity of the retained covariates was investigated, and no issue was identified. All the models were also center-adjusted (baseline hazard stratification for the Cox regressions). Statistical analyses were performed using Plug-Stat© (www.labcom-risca.com).

## Results

### Cohort description

The characteristics of the 4691 patients included in the study are described in Tables 1 and 2. Three thousand nine hundred forty-four patients were in the non-obese group (NOG, 84.1%) versus 747 in the obese group (OG, 15.9%). One hundred thirteen patients had a BMI > 35 kg/m^2^. In the NOG, the mean BMI was 24.2 (± 2.9) kg/m^2^ (ranging from 18.6 to 30.0) versus 32.8 (± 2.5) kg/m^2^ in the OG (ranging from 30.1 to 50.3). As expected, several characteristics at transplantation differed between the two groups. The prevalence of recipients older than 55 years was 60.4% in the OG versus 50.3% in the NOG (*p* < 0.0001). The percentage of male recipients was lower in the OG (55.4% versus 65.0, *p* < 0.0001), but the prevalence of patients with diabetes and history of dyslipidemia was higher (38.3% versus 16.2%, *p* < 0.0001; and 56.0% versus 38.4%, *p* < 0.0001). Obese patients were more likely to receive ECD grafts (52.3% versus 46.1%, *p* = 0.0021).

**Table 1:** Description of the recipients’ characteristics according to obesity status

	Whole sample (*n* = 4691)			Non-obese (*n* = 3944)			Obese (*n* = 747)			*p*-value
	NA	n	%	NA	n	%	NA	n	%	
**Male**	0	2977	63.5	0	2563	65.0	0	414	55.4	< 0.0001
Recurrent causal nephropathy (*)	1	1114	23.8	1	983	24.9	0	131	17.5	< 0.0001
**Causal nephropathy**	1			1			0			< 0.0001
**Diabetes**		569	12.1		392	9.9		177	23.6	
**Glomerulopathy**		935	19.8		823	20.8		112	15.0	
**Other**		2252	47.8		1963	49.5		289	38.6	
**Tubulointerstitial nephritis**		414	8.8		352	8.9		62	8.3	
**Vascular disease**		540	11.5		431	10.9		109	14.6	
**Preemptive transplantation**	8	450	9.6	8	395	10.0	0	55	7.4	0.0231
**Peritoneal dialysis**	8	454	9.7	8	398	10.1	0	55	7.4	0.0240
**History of diabetes**	0	923	19.7	0	637	16.2	0	286	38.3	< 0.0001
**History of hypertension**	0	3949	84.2	0	3301	83.7	0	648	86.7	0.0362
**History of vascular disease**	0	837	17.8	0	696	17.6	0	141	18.9	0.4214
**History of cardiac disease**	0	1355	28.9	0	1095	27.8	0	260	34.8	0.0001
**History of cardiovascular disease**	0	1806	38.5	0	1475	37.4	0	331	44.3	0.0004
**History of malignancy**	0	476	10.1	0	406	10.3	0	70	9.4	0.4435
**History of dyslipidemia**	0	1934	41.2	0	1516	38.4	0	418	56.0	< 0.0001
**History of B or C hepatitis**	0	250	5.3	0	224	5.7	0	26	3.5	0.0142
**Positive CMV serology**	47	3028	65.2	40	2515	64.4	7	513	69.3	0.0102
**Positive EBV serology**	62	4488	97.0	53	3765	96.8	9	723	98.0	0.0806
**Positive class I anti-HLA antibodies**	378	1369	31.7	318	1145	31.6	60	224	32.6	0.5956
**Positive class II anti-HLA antibodies**	432	1223	28.7	360	1033	28.8	72	190	28.1	0.7224
**Blood group**	3			1			2			0.4660
**A**		2018	43.0		1695	43.0		323	43.4	
**AB**		221	4.7		178	4.5		43	5.8	
**B**		533	11.4		448	11.4		85	11.4	
**O**		1916	40.9		1622	41.1		294	39.5	
**HLA A-B-DR incompatibilities ≥ 4**	43	720	15.5	34	610	15.6	9	110	14.9	0.6318
**ABO incompatible transplants**	0	1	0.1	0	6	0.2	0	1	0.1	1.0000
**Depleting induction**	0	2505	53.4	0	2060	52.2	0	445	59.6	0.0002
**Maintenance therapy**										
**Calcineurin inhibitors**	0	4611	97.9	0	3873	97.8	0	738	98.5	0.2240
**Corticosteroids**	0	4832	93.0	0	3683	93.0	0	699	93.3	0.7775
**Age ≥ 55 years**	0	2435	51.9	0	1984	50.3	0	451	60.4	< 0.0001
	**NA**	**mean**	**sd**	**NA**	**mean**	**sd**	**NA**	**mean**	**sd**	***p*-value**
**Waiting list (months)**	108	25.9	22.7	91	25.9	22.8	17	25.6	21.7	0.6865
**Age (years)**	0	53.5	13.2	0	52.9	13.5	0	56.2	11.2	< 0.0001
**Cold ischemia time (hours)**	18	18.1	7.4	13	18.1	7.5	5	18.3	7.1	0.4490

*Abbreviations*: *HLA* Human Leucocyte Antigen, *CMV* Cytomegalovirus, *EBV* Epstein-Barr Virus

**Table 2:** Description of the donors’ characteristics according to obesity status

	Whole sample (*n* = 4691)			Non-obeses (*n* = 3944)			Obeses (*n* = 747)			*p*-value
	NA	n	%	NA	n	%	NA	n	%	
**Male**	5	2766	59.0	3	2320	58.9	2	446	59.9	0.6117
**ECD**	53	2186	47.1	48	1798	46.1	5	388	52.3	0.0021
**Non heart-beating**	0	204	4.3	0	180	4.6	0	24	3.2	0.0969
**Cerebro-Vascular cause of death (Stroke or bleeding)**	12	2533	54.1	10	2105	53.5	2	428	57.4	0.0477
**Hypertension**	192	1409	31.3	166	1174	31.1	26	235	32.6	0.4203
**Positive CMV serology**	13	2584	55.2	11	2165	55.0	2	419	56.2	0.5477
**Positive EBV serology**	42	4461	96.0	34	3743	95.7	8	718	97.2	0.0704
**Blood group**	3			3			0			0.5152
**A**		2021	43.1		1696	43.0		325	43.5	
**AB**		195	4.2		157	4.0		38	5.1	
**B**		486	10.4		408	10.4		78	10.4	
**O**		1986	42.4		1680	42.6		306	41.0	
**Age ≥ 55 years**	15	2509	53.7	14	2062	52.5	1	447	59.9	0.0002
	**NA**	**mean**	**sd**	**NA**	**mean**	**sd**	**NA**	**mean**	**sd**	***p*-value**
**Last serum creatinine (**μmol**/l)**	29	92.5	58.8	24	92.1	57.7	5	94.6	64.0	0.3093
**Age (years)**	15	54.1	22.1	14	53.6	23.2	1	56.8	14.3	< 0.0001

*Abbreviations*: *ECD* Expanded Donor Criteria, *CMV* Cytomegalovirus, *EBV* Epstein-Barr Virus

During the follow-up, 462 patients died with a functioning graft (including 101 in the OG), 614 returned to dialysis (including 118 in the OG) and 12 were preemptive re-transplantations (including 1 in the OG). Median follow-up time for the cohort was 4.0 years (range from 0.0 to 13.2).

### Graft and/or patient survival

**Fig. 1:**
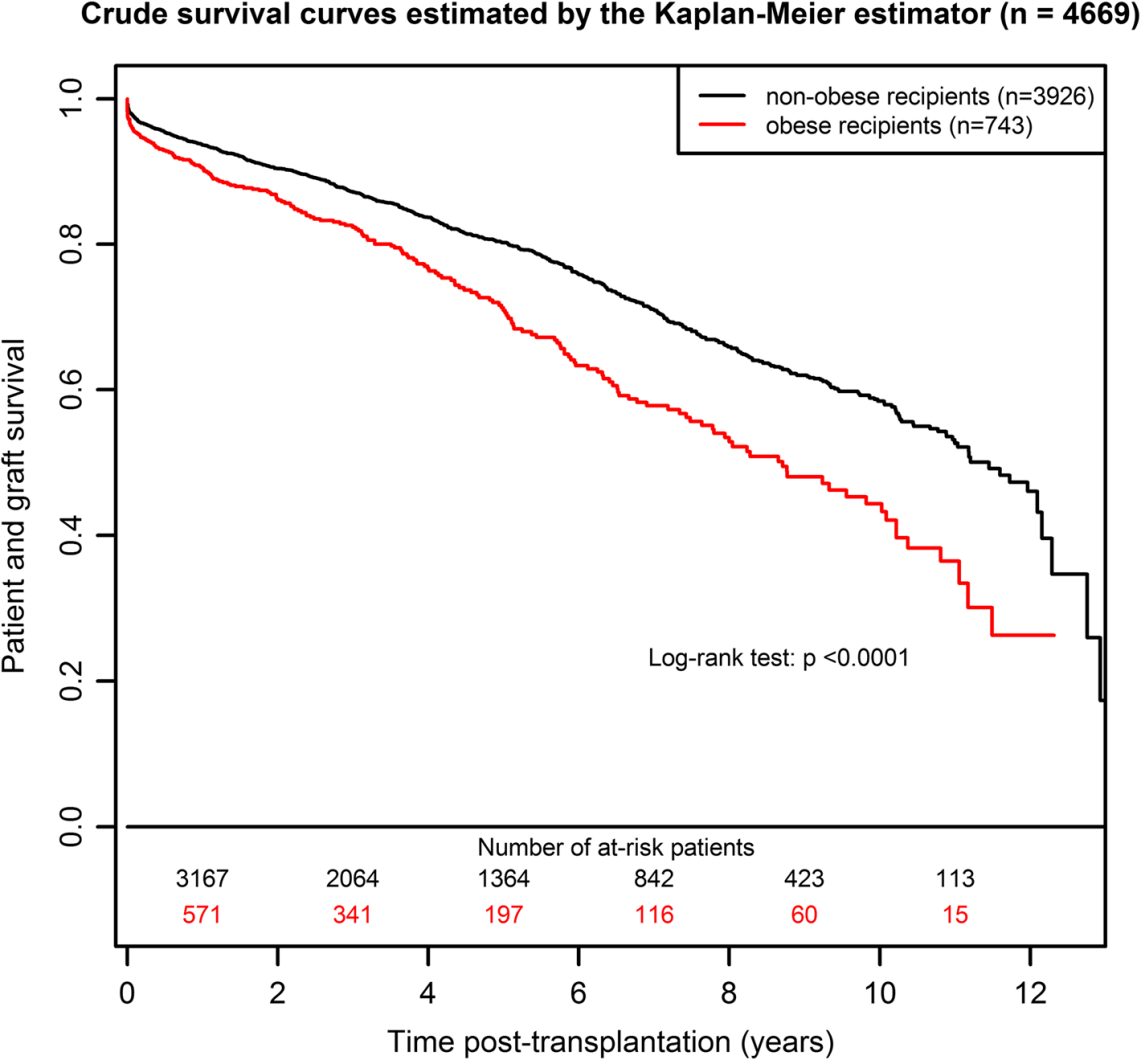
Patient and graft survival curves according to obesity status

**Fig. 2:**
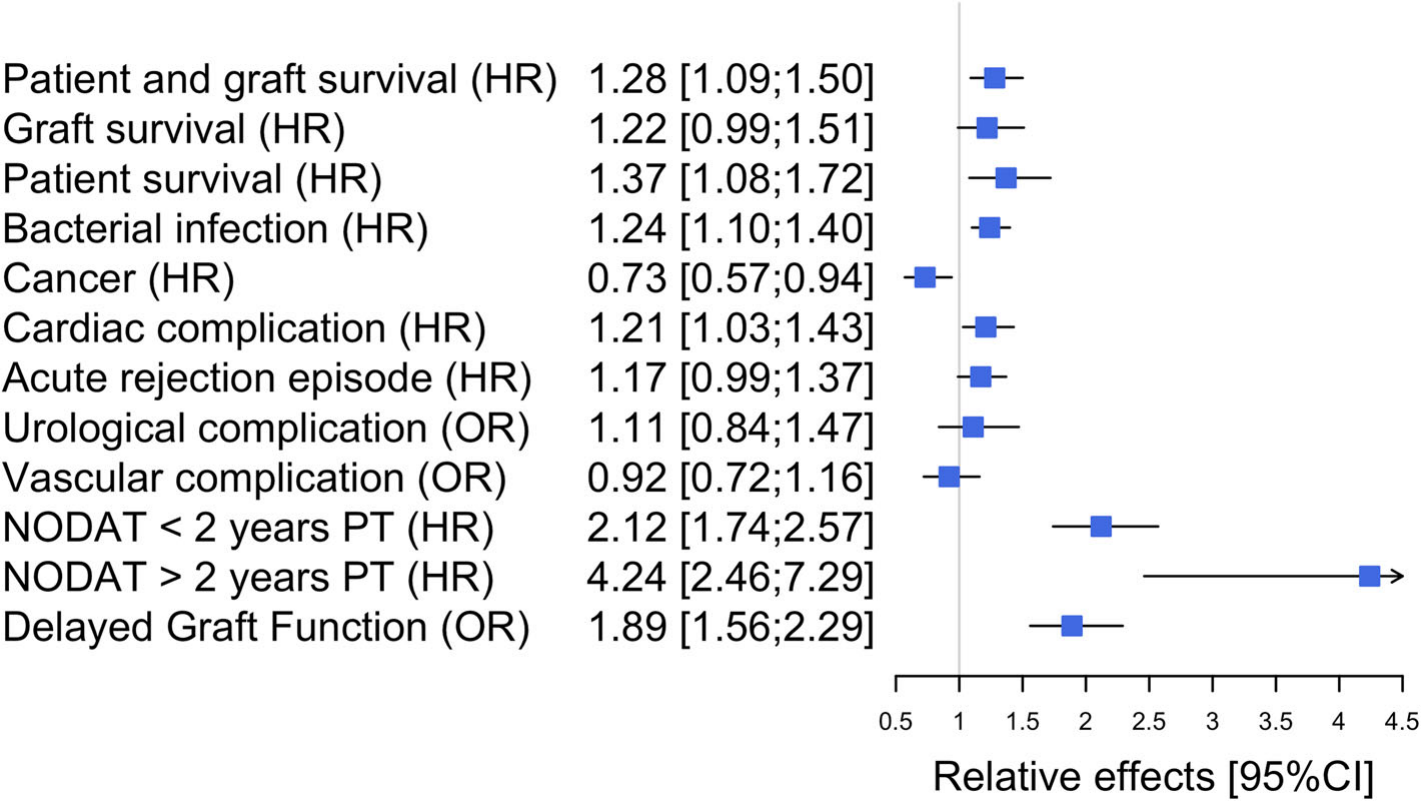
Summary of adjusted relative effects of obese versus non-obese recipients

The patient and graft survival curves are presented in Fig. 1. The survival was 44% (95%CI from 38 to 52%) at 10 years post-transplantation in the OG versus 58% (95%CI from 55 to 61%) in the NOG. As illustrated in Table S1, the corresponding adjusted HR (Hazard Ratio) of the obese versus non-obese group was 1.28 (95%CI from 1.09 to 1.50, *p* = 0.0021): an obese patient has a 1.28-fold increased risk of death or return to dialysis compared to a non-obese patient with similar risk factors at transplantation. When deaths were censored, the adjusted HR of graft failure was 1.22 (Table S2, 95%CI from 0.99 to 1.51, *p* = 0.0666). When graft failures were censored, the adjusted HR of death with a functioning graft was 1.37 (Table S3, 95%CI from 1.08 to 1.72, *p* = 0.0086). These two cause-specific results illustrate that the worse prognosis for obese recipients in terms of patient and graft survival was related to excess mortality. All the results in terms of adjusted relative effects are summarized in Fig. 2.

In addition, we performed the following comparisons to evaluate if 30 kg/m^2^ corresponded to a sudden threshold in the risk of graft failure: < 25.0 versus 25.0–27.5, 25.0–27.5 versus 27.5–30.0, 27.5–30.0 versus 30.0–32.5, and 30.0–32.5 versus > 32.5 kg/m2. As shown in Figure S1, no such threshold was identified. Finally, we explored the change in the obesity effect according to the recipient age (Figure S2). In recipients < 55 years old, the HR of obese versus non-obese patients was 1.06 (95%CI from 0.77 to 1.45), 1.04 (95%CI from 0.73 to 1.47), and 1.10 (95%CI from 0.60 to 2.03) for patient and graft, graft, and patient survival, respectively. The corresponding estimations were 1.40 (95%CI from 1.17 to 1.67), 1.26 (95%CI from 0.96 to 1.65), and 1.46 (95%CI from 1.15 to 1.87), highlighting the higher impact of obesity in older recipients.

### Bacterial infection, neoplasia, cardiac complications and acute rejection episodes

The cumulative probability of bacterial infection at 5 years post-transplantation was 0.44 (95%CI from 0.42 to 0.46) in the NOG versus 0.55 (95%CI from 0.50 to 0.58) in the OG. The corresponding adjusted HR was 1.24 (Table S4, 95%CI from 1.10 to 1.40, *p* = 0.0006), meaning a 1.2-fold increase in the risk of bacterial infection for obese patients. The cumulative probability of cancer at 5 years post-transplantation was 0.16 (95%CI from 0.15 to 0.18) in the NOG versus 0.14 (95%CI from 0.11 to 0.18) in the OG. In contrast to bacterial infections, obese patients had a lower risk of cancer compared with non-obese patients (Table S5, adjuster HR = 0.73, 95%CI from 0.57 to 0.94, *p* = 0.0160). The cumulative probability of cardiac complication at 5 years post-transplantation was 0.23 (95%CI from 0.22 to 0.25) in the NOG versus 0.32 (95%CI from 0.28 to 0.36) in the OG. The corresponding adjusted HR was 1.21 (Table S6, 95%CI from 1.03 to 1.43, *p* < 0.0192). Finally, the cumulative probability of an acute rejection episode at 5 years post-transplantation was 0.28 (95%CI from 0.26 to 0.29) in the NOG versus 0.31 (95%CI from 0.27 to 0.35) in the OG. The corresponding adjusted HR for obese versus non-obese recipients was 1.17 (Table S7, 95%CI from 0.99 to 1.37, *p* = 0.0580).

### Early urological surgical and vascular complications

The overall percentage of urological surgical complications was 8.6% (*n* = 402): 9.9% in the OG (95%CI from 9.0 to 10.8%) versus 8.3% in the NOG (95%CI from 7.5 to 9.2%). When adjusted on possible confounders (Table S8a), no significant difference was identified (OR = 1.11, 95%CI from 0.84 to 1.47, *p* = 0.4443). However, we observed that obese patients presented higher rates of lymphocele that required surgical intervention (48.6%) compared to non-obese patients (36%). In contrast, we observed less ureteral fistula in obese patients (23% versus 35.1% respectively, Table S8b).

Vascular complications were similar for the two groups, with 15.5% in the OG (95%CI from 14.4 to 16.7%) versus 14.8% in the NOG (95%CI from 13.7 to 15.9%). Adjusted results confirmed non-significant differences in vascular complications within the first month after the transplantation (Table S9a, OR = 0.92, 95%CI from 0.72 to 1.16). As illustrated in Table S9b, despite overall vascular complications being quite similar in the two groups, we observed that arterial thrombosis was more frequent in obese (23.3%) compared to non-obese patients (11.6%).

### Subgroup analyses: metabolic complication and DGF

For NODAT analysis, 930 patients were excluded because they were already diabetic before the transplantation. Characteristics of the 3760 studied patients are described in Table S10. Four hundred fifty-eight patients were in the OG (12.2%) versus 3302 in the NOG (87.8%). The cumulative probability of developing NODAT at 5 years post-transplantation was 0.17 (95%CI from 0.16 to 0.19) in the NOG compared to 0.42 in the OG (95%CI from 0.36 to 0.47). After considering possible confounders (Table S11), obese patients presented a 1.2-fold increase in the risk of NODAT within 2 years after transplantation (95%CI from 1.74 to 2.57, *p* < 0.0001). After 2 years post-transplantation, this hazard ratio increased to 4.24 (95%CI from 2.46 to 7.29, *p* < 0.0001).

For DGF analysis, 1005 patients were excluded because they were undergoing peritoneal dialysis or not dialyzed before transplantation, the need for dialysis in the first week post-transplantation being not evaluable for these recipients. Characteristics of the 3686 patients are described in Table S12. Six hundred fifteen patients were in the OG (16.7%) versus 3071 in the NOG (83.3%). We found a DGF prevalence of 26.2% (95%CI from 24.8 to 27.6%) in the NOG compared to 42.1% (95%CI from 40.5 to 43.7%) in the OG. When adjusted for possible confounders (Table S13), we confirmed that obese patients have a higher DGF susceptibility (OR = 1.89; 95%CI from 1.56 to 2.29; *p* < 0.0001) compared to non-obese patients.

## Discussion

Based on a French cohort, we report that obesity does not significantly increase the risk of urologic or vascular complications or graft loss, but seems to increase the risk of cardiac and infectious complications and the mortality.

Our results are likely to be representative of the entire French transplantation cohort since we studied more than 4500 recipients of a first kidney graft between 2005 and 2016, from a multicenter cohort gathering one third of the national transplantation activity. The proportion of obese patients in our transplantation cohort was 15.9%, in-line with French practices recently described from the national French Registry. One can note that 20% of dialyzed patients in France are obese, illustrating that obesity may be an obstacle to transplantation access, as previously reported [20]. This was the main reason of our study; obese patients have a lower access to transplantation in France, whilst the risk/benefit ratio associated with transplantation remains unknown in French obese patients. The French health authority recommends limiting transplantation to recipients with a BMI below 35 kg/m^2^ [21], and this cutoff is mainly based on North-American studies. In our cohort, only 2.4% (*n* = 113) of patients presented a BMI above 35 kg/m^2^, meaning that the relevance of this threshold cannot be investigated due to the small sample size.

Concerning the patient demographic characteristics, we observed that obese patients presented a different profile at transplantation since they were more likely to be female, older, or with higher rates of diabetes and dyslipidemia. This could explain why they were more likely to receive an ECD donor kidney. These characteristics were considered as potential confounders in the multivariate models.

In terms of early complications, and particularly delayed graft function, our results agree with previously published findings [16, 22]. DGF is the most consensual complication observed in obese patients. This could be explained by the greater difficulty in assessing the need for dialysis in these overweight patients, resulting in an over-indication for dialysis post-transplantation. Another explanation could be a longer surgery time required for kidney implantation in obese patients which may predispose to additional complications [23]. This was not the case in our cohort since we reported similar risk of urologic and vascular complications in obese and non-obese patients. However, we only retained early complications requiring surgical intervention, which probably underestimated these events.

We also observed a trend for a higher rate of acute rejections in obese patients, possibly explained by the higher DGF rate [24], the increase in inflammation and alloimmunity and the decrease in the bioavailability of immunosuppression [25]. In addition, the increased incidence of DGF and acute rejection are in agreement with the lower patient and graft survival we observed in the obese patients. Other explanations for the increased risk of death with a functioning graft and higher risk of graft failure for the obese group are the higher incidence of NODAT, more serious bacterial infectious diseases and more cardiac complications.

We investigated whether the risks of graft failure and/or death for obese patients increased steadily with BMI. Whilst we did not observe such a trend, this may be due to sample-to-sample fluctuations caused by the small number of patients with a very high BMI. We also explored the interaction between the recipient age and BMI. In contrast to the lack of a deleterious effect of obesity observed for older patients under dialysis [17], we observed a greater risk of death and patient and graft failure due to obesity in older kidney transplant recipients.

Two North-American studies reported a beneficial effect of transplantation for obese patients with 3.3% deaths per year compared to 6.6% for those who stayed on the waiting list [26, 27]. Therefore, despite our finding that obesity correlates with worse short and long-term graft survival and an increased risk of death after transplantation, we could not conclude that it is preferable to maintain these patients on dialysis. Nevertheless, our results reinforce the potential benefit of helping obese transplant candidates lose weight before transplantation, for instance by using bariatric surgery [28]. Dietary intervention for obese patients with a lower BMI before transplantation remains debatable [29].

Other limitations of our study can be outlined. Firstly, obesity is also the consequence of crucial but uncontrollable factors in our cohort, including genetic considerations, social status, eating, physical activity habits and stress, which could obviously limit the interpretation of our results. Secondly, the BMI per se is a rough marker of obesity. Thirdly, additional outcomes would have been interesting to report, such as length of hospital stay or wound complications. Fourthly, for the analyses related to the DGF, we excluded the patients previously on peritoneal dialysis. This choice can be debated [30]. These patients could need dialysis in the week after the transplantation, which could be considered as delayed graft function. However, these patients often present preserved diuresis and residual glomerular filtration rate, which is associated with a lower prevalence of the DGF in this population. In contrast, because the dialysis is easier to perform in this population, it is more frequently use for their comfort. Finally, using BMI to define obesity could also be another limitation of our study, abdominal circumference or other morphometric models could be more helpful to define obesity from a surgical point of view [31]. In addition, this study did not include any robotic transplantation. This mini-invasive approach is nowadays well established, even in European centres, to decrease abdominal wound complications in obese patients [32].

## Conclusions

In conclusion, our study provides updated results related to outcomes of French obese kidney transplant recipients. French obese patients presented a higher risk of death, serious infections and cardiac complications but not early urologic or vascular complications. The prognosis is even worse for patients who are both obese and elderly. In order to improve graft allocation procedures, epidemiological studies aimed at comparing obese kidney transplant recipients versus obese candidates staying on dialysis are needed, with specific attention to the recipient and donor characteristics that can interact with the transplantation effect.

## Supplementary Information

**Additional file 1.**

## Data Availability

Data from the DIVAT cohort is not publicly available. The complete procedure is explained at the DIVAT web site (http://www.divat.fr/access-to-data). Briefly, the first step is to complete a collaboration form. The scientific committee then studies the project and offers a response within 30 days. If agreed, the anonymized database is sent. Otherwise, an update of the request can be referred to the board. This process may be free of charge for academic studies.

## Abbreviations

BMI: Body mass index
CI: Confidence interval
CMV: Cytomegalovirus
DGF: Delayed graft function
DIVAT: Données Informatisées et VAlidées en Transplantation
n: Effective
ESRD: End-stage renal disease
EBV: Epstein-Barr Virus
ECD: Expanded criteria donor
HR: Hazard ratio
HLA: Human Leucocyte Antigen
m: Mean
NA: Number of missing values
NODAT: New Onset Diabetes After Transplantation
NOG: Non-obese group
OG: Obese group
OR: Odds-ratio
HR: Hazards ratio
PTLD: Post-Transplant Lymphoproliferative Disease
sd: Standard deviation
