# Supplementary material for: Renal transplantation outcomes in obese patients: a French cohort-based study
Source: BMC Nephrol. 2021 Mar 5;22:79. doi: 10.1186/s12882-021-02278-1 (PMC7934368; doi:10.1186/s12882-021-02278-1)

**Supplementary data**

**TABLE S1**. Results of the multivariable Cox model studying the risk of death or graft failure (62 patients were excluded because missing data).

|  | **HR** | **95% CI** | **p-value** |
| --- | --- | --- | --- |
| **Obese recipient** | 1.28 | [1.09 ; 1.50] | 0.0021 |
| **Male recipient** | 1.12 | [0.98 ; 1.28] | 0.0895 |
| **Recurrent causal nephropathy** | 1.06 | [0.91 ; 1.23] | 0.4740 |
| **Preemptive transplantation** | 0.68 | [0.54 ; 0.86] | 0.0016 |
| **History of diabetes** | 1.43 | [1.23 ; 1.66] | <0.0001 |
| **History of cardiovascular disease** | 1.58 | [1.39 ; 1.80] | <0.0001 |
| **History of dyslipemia** | 1.15 | [1.01 ; 1.31] | 0.0302 |
| **ECD donor** | 1.54 | [1.33 ; 1.79] | <0.0001 |
| **Depleting induction** | 0.95 | [0.82 ; 1.10] | 0.4686 |
| **Recipient age ≥55 years** | 1.45 | [1.24 ; 1.70] | <0.0001 |

**TABLE S2**. Results of the multivariable Cox model studying the risk of death censored graft failure (61 patients were excluded because missing data).

|  | **HR** | **95% CI** | **p-value** |
| --- | --- | --- | --- |
| **Obese recipient** | 1.22 | [0.99 ; 1.51] | 0.0666 |
| **Male recipient** | 1.09 | [0.92 ; 1.29] | 0.3123 |
| **Preemptive transplantation** | 0.65 | [0.47 ; 0.90] | 0.0084 |
| **History of diabetes** | 1.27 | [1.04 ; 1.55] | 0.0209 |
| **History of cardiovascular disease** | 1.41 | [1.19 ; 1.67] | 0.0001 |
| **History of dyslipemia** | 1.25 | [1.06 ; 1.48] | 0.0082 |
| **ECD donor** | 1.84 | [1.50 ; 2.24] | <0.0001 |
| **Non-heart-beating-donor** | 2.06 | [1.38 ; 3.07] | 0.0004 |
| **Recipient age ≥55 years** | 0.94 | [0.77 ; 1.15] | 0.5405 |

**TABLE S3**. Results of the multivariable Cox model studying the patient survival (returns in dialysis were censored, 62 patients were excluded because missing data).

|  | **HR** | **95% CI** | **p-value** |
| --- | --- | --- | --- |
| **Obese recipient** | 1.37 | [1.08 ; 1.72] | 0.0086 |
| **Male recipient** | 1.14 | [0.93 ; 1.39] | 0.2177 |
| **Recurrent causal nephropathy** | 0.91 | [0.71 ; 1.19] | 0.4988 |
| **Preemptive transplantation** | 0.68 | [0.48 ; 0.98] | 0.0379 |
| **History of diabetes** | 1.54 | [1.25 ; 1.91] | 0.0001 |
| **History of cardiovascular disease** | 1.78 | [1.45 ; 2.18] | <0.0001 |
| **History of dyslipemia** | 0.99 | [0.81 ; 1.20] | 0.9079 |
| **ECD donor** | 1.00 | [0.79 ; 1.26] | 0.9959 |
| **Depleting induction** | 0.91 | [0.72 ; 1.14] | 0.4013 |
| **Recipient age (years)** | 1.07 | [1.05 ; 1.08] | <0.0001 |

TABLE S4. Results of the multivariable Cox model studying the risk of bacterial infection (62 patients were excluded because missing data).

|  | **HR** | **95% CI** | **p-value** |
| --- | --- | --- | --- |
| **Obese recipient** | 1.24 | [1.10 ; 1.40] | 0.0006 |
| **Male recipient** | 0.68 | [0.62 ; 0.75] | <0.0001 |
| **Recurrent causal nephropathy** | 0.93 | [0.83 ; 1.05] | 0.2358 |
| **Preemptive transplantation** | 0.91 | [0.77 ; 1.07] | 0.2303 |
| **History of diabetes** | 1.09 | [0.96 ; 1.22] | 0.1857 |
| **History of hypertension** | 0.86 | [0.76 ; 0.98] | 0.0220 |
| **History of cardiovascular disease** | 1.17 | [1.06 ; 1.30] | 0.0016 |
| **History of dyslipemia** | 0.98 | [0.89 ; 1.08] | 0.7026 |
| **History of B or C hepatitis** | 1.29 | [1.05 ; 1.58] | 0.0157 |
| **ECD donor** | 1.22 | [1.09 ; 1.37] | 0.0007 |
| **Depleting induction** | 1.03 | [0.92 ; 1.14] | 0.6482 |
| **Recipient age (years)** | 1.01 | [1.00 ; 1.01] | 0.0001 |

TABLE S5. Results of the multivariable Cox model studying the risk of cancer (neoplastic, squamous cell or lymphoma cancer, 114 patients were excluded because of missing data).

|  | **HR** | **95% CI** | **p-value** |
| --- | --- | --- | --- |
| **Obese recipient** | 0.73 | [0.57 ; 0.94] | 0.0160 |
| **Male recipient** | 1.46 | [1.22 ; 1.75] | <0.0001 |
| **History of hypertension** | 1.13 | [0.87 ; 1.47] | 0.3547 |
| **History of cardiovascular disease** | 1.03 | [0.87 ; 1.22] | 0.7390 |
| **History of malignancy** | 1.72 | [1.38 ; 2.15] | <0.0001 |
| **Positive recipient EBV serology** | 0.60 | [0.40 ; 0.89] | 0.0124 |
| **ECD donor** | 1.09 | [0.88 ; 1.34] | 0.4255 |
| **Recipient age (years)** | 1.05 | [1.04 ; 1.06] | <0.0001 |

TABLE S6. Results of the multivariable Cox model studying the risk of cardiac complication (62 patients were excluded because of missing data).

|  | **HR** | **95% CI** | **p-value** |
| --- | --- | --- | --- |
| **Obese recipient** | 1.21 | [1.03 ; 1.43] | 0.0192 |
| **Male recipient** | 1.03 | [0.91 ; 1.18] | 0.6190 |
| **Recurrent causal nephropathy** | 0.95 | [0.80 ; 1.12] | 0.5223 |
| **Preemptive transplantation** | 0.83 | [0.65 ; 1.06] | 0.1342 |
| **History of hypertension** | 1.08 | [0.88 ; 1.31] | 0.4678 |
| **History of cardiovascular disease** | 1.94 | [1.70 ; 2.22] | <0.0001 |
| **History of dyslipemia** | 0.94 | [0.83 ; 1.08] | 0.3871 |
| **ECD donor** | 1.11 | [0.95 ; 1.29] | 0.2014 |
| **Recipient age (years)** | 1.03 | [1.02 ; 1.04] | <0.0001 |

TABLE S7. Results of the multivariable Cox model studying the risk of acute rejection episode (98 patients were excluded because missing data).

|  | **HR** | **95% CI** | **p-value** |
| --- | --- | --- | --- |
| **Obese recipient** | 1.17 | [0.99 ; 1.37] | 0.0580 |
| **Recurrent causal nephropathy** | 1.15 | [1.00 ; 1.32] | 0.0439 |
| **History of diabetes** | 1.20 | [1.03 ; 1.41] | 0.0234 |
| **History of hypertension** | 1.16 | [0.98 ; 1.37] | 0.0933 |
| **Positive recipient CMV serology** | 1.13 | [0.99 ; 1.28] | 0.0670 |
| **ECD donor** | 1.38 | [1.19 ; 1.60] | <0.0001 |
| **Depleting induction** | 0.70 | [0.61 ; 0.79] | <0.0001 |
| **Recipient age (years)** | 0.98 | [0.98 ; 0.99] | <0.0001 |

TABLE S8a. Results of the multivariable logistic model of urological complications (104 patients were excluded because missing data).

|  | **OR** | **95% CI** | **p-value** |
| --- | --- | --- | --- |
| **Obese recipient** | 1.11 | [0.84 ; 1.47] | 0.4443 |
| **Male recipient** | 1.34 | [1.07 ; 1.68] | 0.0121 |
| **Preemptive transplantation** | 0.64 | [0.41 ; 0.99] | 0.0440 |
| **History of diabetes** | 1.00 | [0.77 ; 1.31] | 0.9718 |
| **History of cardiovascular disease** | 1.24 | [0.99 ; 1.55] | 0.0590 |
| **Positive recipient CMV serology** | 1.11 | [0.88 ; 1.40] | 0.3649 |
| **ECD donor** | 1.44 | [1.10 ; 1.90] | 0.0089 |
| **Non-heart-beating-donor** | 0.61 | [0.31 ; 1.19] | 0.1452 |
| **Recipient age (years)** | 1.00 | [0.99 ; 1.02] | 0.3873 |

TABLE S8b. Descriptive table of urological complication (at least one) occurring within the first 30 days after the surgery.

|  | **Whole sample (n=402)** | | **Non-obeses (n=328)** | | | **Obeses (n=74)** | |
| --- | --- | --- | --- | --- | --- | --- | --- |
|  | **n** | **%** | | **n** | **%** | **n** | **%** |
| **Collection with required drainage** | 53 | 13.2 | | 42 | 12.8 | 11 | 14.9 |
| **lymphocele with drainage or surgery resumption** | 154 | 38.3 | | 118 | 36.0 | 36 | 48.6 |
| **Vesicoureteral reflux on the graft** | 2 | 0.5 | | 2 | 0.6 | 0 | 0.0 |
| **Ureteral stenosis** | 61 | 15.2 | | 51 | 15.5 | 10 | 13.5 |
| **Urinoma (fistula or uretero-vesical necrosis)** | 132 | 32.8 | | 115 | 35.1 | 17 | 23.0 |

TABLE S9a. Results of the multivariable logistic model of vascular system complications (128 patients were excluded because missing data).

|  | **OR** | **95% CI** | **p-value** |
| --- | --- | --- | --- |
| **Obese recipient** | 0.92 | [0.72 ; 1.16] | 0.4628 |
| **Preemptive transplantation** | 0.89 | [0.64 ; 1.25] | 0.4982 |
| **History of diabetes** | 1.07 | [0.86 ; 1.33] | 0.5678 |
| **History of hypertension** | 1.15 | [0.89 ; 1.48] | 0.2924 |
| **History of cardiovascular disease** | 1.39 | [1.16 ; 1.67] | 0.0004 |
| **History of B or C hepatitis** | 1.27 | [0.90 ; 1.79] | 0.1797 |
| **Positive recipient CMV serology** | 1.31 | [1.08 ; 1.60] | 0.0059 |
| **Positive recipient EBV serology** | 0.62 | [0.40 ; 0.98] | 0.0388 |
| **ECD donor** | 1.16 | [0.93 ; 1.43] | 0.1809 |
| **Recipient age (years)** | 1.00 | [0.99 ; 1.01] | 0.7939 |

TABLE S9b. Descriptive table of types of complications according to exposure of interest in patients with at least one vascular system complications.

|  | **Whole sample (n=700)** | | **Non-obeses (n=584)** | | **Obeses (n=116)** | |
| --- | --- | --- | --- | --- | --- | --- |
|  | **n** | **%** | **n** | **%** | **n** | **%** |
| **Arterial or arteriovenous aneurysm** | 2 | 0.3 | 2 | 0.3 | 0 | 0.0 |
| **Arteriovenous fistula** | 7 | 1.0 | 7 | 1.2 | 0 | 0.0 |
| **Operating site hemorrhage** | 458 | 65.4 | 389 | 66.6 | 69 | 59.5 |
| **Arterial graft pathology** | 88 | 12.6 | 80 | 13.7 | 8 | 6.9 |
| **Partial or total arterial thrombosis of the graft** | 95 | 13.6 | 68 | 11.6 | 27 | 23.3 |
| **Partial or total venous thrombosis** | 50 | 7.1 | 38 | 6.5 | 12 | 10.3 |

TABLE S10. Description of the patients studied for the metabolic complication analysis according to the obesity status.

|  | **Whole sample (n=3760)** | | | **Non-obeses (n=3302)** | | | **Obeses (n=458)** | | | **p-value** |
| --- | --- | --- | --- | --- | --- | --- | --- | --- | --- | --- |
|  | **NA** | **n** | **%** | **NA** | **n** | **%** | **NA** | **n** | **%** |  |
| **Male recipient** | 0 | 2348 | 62.4 | 0 | 2110 | 63.9 | 0 | 238 | 52.0 | <0.0001 |
| **Recurrent causal nephropathy** | 0 | 1037 | 27.6 | 0 | 933 | 28.3 | 0 | 104 | 22.7 | 0.0128 |
| **Preemptive transplantation** | 7 | 387 | 10.3 | 7 | 350 | 10.6 | 0 | 37 | 8.1 | 0.0935 |
| **History of hypertension** | 0 | 3121 | 83.0 | 0 | 2727 | 82.6 | 0 | 394 | 86.0 | 0.0662 |
| **History of cardiovascular disease** | 0 | 1261 | 33.5 | 0 | 1094 | 33.1 | 0 | 167 | 36.5 | 0.1570 |
| **History of malignancy** | 0 | 385 | 10.2 | 0 | 341 | 10.3 | 0 | 44 | 9.6 | 0.6338 |
| **History of dyslipemia** | 0 | 1376 | 36.6 | 0 | 1151 | 34.9 | 0 | 225 | 49.1 | <0.0001 |
| **History of B or C hepatitis** | 0 | 190 | 5.1 | 0 | 176 | 5.3 | 0 | 14 | 3.1 | 0.0374 |
| **Positive recipient CMV serology** | 42 | 2333 | 62.7 | 37 | 2033 | 62.3 | 5 | 300 | 66.2 | 0.1024 |
| **Positive recipient EBV serology** | 55 | 3580 | 96.6 | 48 | 3141 | 96.5 | 7 | 439 | 97.3 | 0.3708 |
| **Positive anti-class I immunization** | 312 | 1080 | 31.3 | 272 | 937 | 30.9 | 40 | 143 | 34.2 | 0.1744 |
| **Positive anti-class II immunization** | 357 | 953 | 28.0 | 309 | 842 | 28.1 | 48 | 111 | 27.1 | 0.6542 |
| **Recipient blood group** | 2 |  |  | 1 |  |  | 1 |  |  | 0.3103 |
| **A** |  | 1592 | 42.4 |  | 1404 | 42.5 |  | 188 | 41.1 |  |
| **AB** |  | 173 | 4.6 |  | 145 | 4.4 |  | 28 | 6.1 |  |
| **B** |  | 428 | 11.4 |  | 371 | 11.2 |  | 57 | 12.5 |  |
| **O** |  | 1565 | 41.6 |  | 1381 | 41.8 |  | 184 | 40.3 |  |
| **Male donor** | 3 | 2251 | 59.9 | 3 | 1964 | 59.5 | 0 | 287 | 62.7 | 0.2002 |
| **ECD donor** | 42 | 1603 | 43.1 | 38 | 1394 | 42.7 | 4 | 209 | 46.0 | 0.1799 |
| **Non-heart-beating-donor** | 0 | 173 | 4.6 | 0 | 157 | 4.8 | 0 | 16 | 3.5 | 0.2273 |
| **Vascular cause of donor death** | 11 | 1966 | 52.4 | 10 | 1720 | 52.2 | 1 | 246 | 53.8 | 0.5258 |
| **Donor hypertension** | 159 | 1070 | 29.7 | 141 | 945 | 29.9 | 18 | 125 | 28.4 | 0.5227 |
| **Positive donor CMV serology** | 10 | 2048 | 54.6 | 8 | 1800 | 54.6 | 2 | 248 | 54.4 | 0.9171 |
| **Positive donor EBV serology** | 36 | 3578 | 96.1 | 31 | 3133 | 95.8 | 5 | 445 | 98.2 | 0.0117 |
| **Donor blood group** | 2 |  |  | 2 |  |  | 0 |  |  | 0.2518 |
| **A** |  | 1596 | 42.5 |  | 1407 | 42.6 |  | 189 | 41.3 |  |
| **AB** |  | 150 | 4.0 |  | 125 | 3.8 |  | 25 | 5.5 |  |
| **B** |  | 395 | 10.5 |  | 341 | 10.3 |  | 54 | 11.8 |  |
| **O** |  | 1617 | 43.0 |  | 1427 | 43.2 |  | 190 | 41.5 |  |
| **HLA-A-B-DR incompatibilities > 4** | 27 | 568 | 15.2 | 21 | 509 | 15.5 | 6 | 59 | 13.1 | 0.1721 |
| **Depleting induction** | 0 | 1969 | 52.4 | 0 | 1692 | 51.2 | 0 | 277 | 60.5 | 0.0002 |
| **Recipient age ≥55 years** | 0 | 1742 | 46.3 | 0 | 1501 | 45.5 | 0 | 241 | 52.6 | 0.0040 |
|  | **NA** | **mean** | **sd** | **NA** | **mean** | **sd** | **NA** | **mean** | **sd** | **p-value** |
| **Duration on waiting list (months)** | 85 | 26.2 | 22.9 | 72 | 26.2 | 23.0 | 13 | 26.3 | 21.9 | 0.9057 |
| **Donor age (years)** | 12 | 52.8 | 16.3 | 11 | 52.5 | 16.6 | 1 | 54.7 | 14.4 | 0.0026 |
| **Donor creatininemia (μmol/l)** | 23 | 93.3 | 60.0 | 20 | 92.7 | 58.5 | 3 | 97.7 | 69.7 | 0.1471 |
| **Cold ischemia time (hours)** | 14 | 18.1 | 7.0 | 11 | 18.0 | 6.9 | 3 | 18.4 | 7.3 | 0.2781 |

**TABLE S11.** Results of the multivariable logistic model studying the risk of new-onset diabetes mellitus after transplantation (104 patients were excluded because missing data).

|  | **HR** | **95% CI** | **p-value** |
| --- | --- | --- | --- |
| **Obese recipient, before 2 years post-transplantation** | 2.12 | [1.74 ; 2.57] | <0.0001 |
| **Obese recipient, from 2 years post-transplantation** | 4.24 | [2.46 ; 7.29] | <0.0001 |
| **Male recipient** | 0.77 | [0.66 ; 0.91] | 0.0015 |
| **Recurrent causal nephropathy** | 0.73 | [0.61 ; 0.89] | 0.0019 |
| **History of hypertension** | 1.81 | [1.39 ; 2.35] | <0.0001 |
| **History of cardiovascular disease** | 1.18 | [1.00 ; 1.39] | 0.0449 |
| **History of dyslipemia** | 1.12 | [0.96 ; 1.32] | 0.1576 |
| **Positive recipient CMV serology** | 1.29 | [1.08 ; 1.52] | 0.0037 |
| **ECD donor** | 1.02 | [0.84 ; 1.23] | 0.8365 |
| **HLA-A-B-DR incompatibilities > 4** | 1.22 | [1.00 ; 1.50] | 0.0519 |
| **Depleting induction** | 1.11 | [0.93 ; 1.32] | 0.2605 |
| **Recipient age ≥55 years** | 1.52 | [1.25 ; 1.85] | <0.0001 |

**TABLE S12.** Description of the patients studied for the delayed graft function analysis according to the obesity status.

|  | **Whole sample (n=3686)** | | | **Non-obeses (n=3071)** | | | **Obeses (n=615)** | | | **p-value** |
| --- | --- | --- | --- | --- | --- | --- | --- | --- | --- | --- |
|  | **NA** | **n** | **%** | **NA** | **n** | **%** | **NA** | **n** | **%** |  |
| **Male recipient** | 0 | 2368 | 64.2 | 0 | 2021 | 65.8 | 0 | 347 | 56.4 | <0.0001 |
| **Recurrent causal nephropathy** | 0 | 871 | 23.6 | 0 | 761 | 24.8 | 0 | 110 | 17.9 | 0.0002 |
| **History of diabetes** | 0 | 777 | 21.1 | 0 | 528 | 17.2 | 0 | 249 | 40.5 | <0.0001 |
| **History of hypertension** | 0 | 3063 | 83.1 | 0 | 2532 | 82.4 | 0 | 531 | 86.3 | 0.0187 |
| **History of cardiovascular disease** | 0 | 1490 | 40.4 | 0 | 1206 | 39.3 | 0 | 284 | 46.2 | 0.0014 |
| **History of malignancy** | 0 | 377 | 10.2 | 0 | 316 | 10.3 | 0 | 61 | 9.9 | 0.7816 |
| **History of dyslipemia** | 0 | 1473 | 40.0 | 0 | 1134 | 36.9 | 0 | 339 | 55.1 | <0.0001 |
| **History of B or C hepatitis** | 0 | 217 | 5.9 | 0 | 194 | 6.3 | 0 | 23 | 3.7 | 0.0132 |
| **Positive recipient CMV serology** | 35 | 2450 | 67.1 | 30 | 2023 | 66.5 | 5 | 427 | 70.0 | 0.0954 |
| **Positive recipient EBV serology** | 48 | 3536 | 97.2 | 41 | 2939 | 97.0 | 7 | 597 | 98.2 | 0.1036 |
| **Positive anti-class I immunization** | 280 | 1110 | 32.6 | 231 | 924 | 32.5 | 49 | 186 | 32.9 | 0.8795 |
| **Positive anti-class II immunization** | 324 | 1009 | 30.0 | 264 | 850 | 30.3 | 60 | 159 | 28.6 | 0.4431 |
| **Recipient blood group** | 2 |  |  | 1 |  |  | 1 |  |  | 0.5924 |
| **A** |  | 1512 | 41.0 |  | 1255 | 40.9 |  | 257 | 41.9 |  |
| **AB** |  | 165 | 4.5 |  | 132 | 4.3 |  | 33 | 5.4 |  |
| **B** |  | 445 | 12.1 |  | 375 | 12.2 |  | 70 | 11.4 |  |
| **O** |  | 1562 | 42.4 |  | 1308 | 42.6 |  | 254 | 41.4 |  |
| **Male donor** | 5 | 2182 | 59.3 | 3 | 1823 | 59.4 | 2 | 359 | 58.6 | 0.6939 |
| **ECD donor** | 42 | 1715 | 47.1 | 39 | 1391 | 45.9 | 3 | 324 | 52.9 | 0.0014 |
| **Non-heart-beating-donor** | 0 | 128 | 3.5 | 0 | 113 | 3.7 | 0 | 15 | 2.4 | 0.1251 |
| **Vascular cause of donor death** | 8 | 2027 | 55.1 | 7 | 1664 | 54.3 | 1 | 363 | 59.1 | 0.0287 |
| **Donor hypertension** | 165 | 1073 | 30.5 | 143 | 885 | 30.2 | 22 | 188 | 31.7 | 0.4759 |
| **Positive donor CMV serology** | 12 | 2052 | 55.9 | 11 | 1696 | 55.4 | 1 | 356 | 58.0 | 0.2445 |
| **Positive donor EBV serology** | 38 | 3507 | 96.1 | 31 | 2915 | 95.9 | 7 | 592 | 97.4 | 0.0839 |
| **Donor blood group** | 3 |  |  | 3 |  |  | 0 |  |  | 0.6616 |
| **A** |  | 1515 | 41.1 |  | 1258 | 41.0 |  | 257 | 41.8 |  |
| **AB** |  | 144 | 3.9 |  | 115 | 3.7 |  | 29 | 4.7 |  |
| **B** |  | 408 | 11.1 |  | 342 | 11.1 |  | 66 | 10.7 |  |
| **O** |  | 1616 | 43.9 |  | 1353 | 44.1 |  | 263 | 42.8 |  |
| **HLA-A-B-DR incompatibilities > 4** | 37 | 563 | 15.4 | 31 | 474 | 15.6 | 6 | 89 | 14.6 | 0.5420 |
| **Depleting induction** | 0 | 2016 | 54.7 | 0 | 1655 | 53.9 | 0 | 361 | 58.7 | 0.0288 |
|  | **NA** | **mean** | **sd** | **NA** | **mean** | **sd** | **NA** | **mean** | **sd** |  |
| **Recipient age (years)** | 0 | 53.4 | 13.2 | 0 | 52.8 | 13.5 | 0 | 56.2 | 11.2 | <0.0001 |
| **Duration on waiting list (months)** | 79 | 27.9 | 23.6 | 63 | 28.0 | 23.8 | 16 | 26.9 | 22.3 | 0.2567 |
| **Donor age (years)** | 10 | 54.2 | 16.1 | 10 | 53.7 | 16.3 | 0 | 56.8 | 14.3 | <0.0001 |
| **Donor creatininemia (μmol/l)** | 23 | 92.5 | 59.5 | 20 | 92.6 | 58.9 | 3 | 92.0 | 62.2 | 0.8417 |
| **Cold ischemia time (hours)** | 5 | 18.3 | 7.6 | 4 | 18.3 | 7.7 | 1 | 18.5 | 7.1 | 0.6093 |

**TABLE S13.** Results of the multivariable logistic model studying delayed graft function (42 patients were excluded because missing data).

|  | **OR** | **95% CI** | **p-value** |
| --- | --- | --- | --- |
| **Obese recipient** | 1.89 | [1.56 ; 2.29] | <0.0001 |
| **Male recipient** | 1.55 | [1.32 ; 1.81] | <0.0001 |
| **History of diabetes** | 1.03 | [0.85 ; 1.24] | 0.7549 |
| **History of hypertension** | 0.87 | [0.71 ; 1.06] | 0.1735 |
| **History of cardiovascular disease** | 1.30 | [1.12 ; 1.52] | 0.0008 |
| **History of dyslipemia** | 0.96 | [0.82 ; 1.12] | 0.5938 |
| **ECD donor** | 1.04 | [0.87 ; 1.26] | 0.6576 |
| **Depleting induction** | 1.15 | [0.97 ; 1.37] | 0.0961 |
| **Recipient age (years)** | 1.00 | [1.00 ; 1.01] | 0.3880 |

**FIGURE S1. BMI-based subgroup analyses to estimate the adjusted hazard ratio (HR) of obese versus non-obese patients for the long-term outcomes: the patient and graft survival (PGS), the graft survival (GS), and the patient survival (PS).**


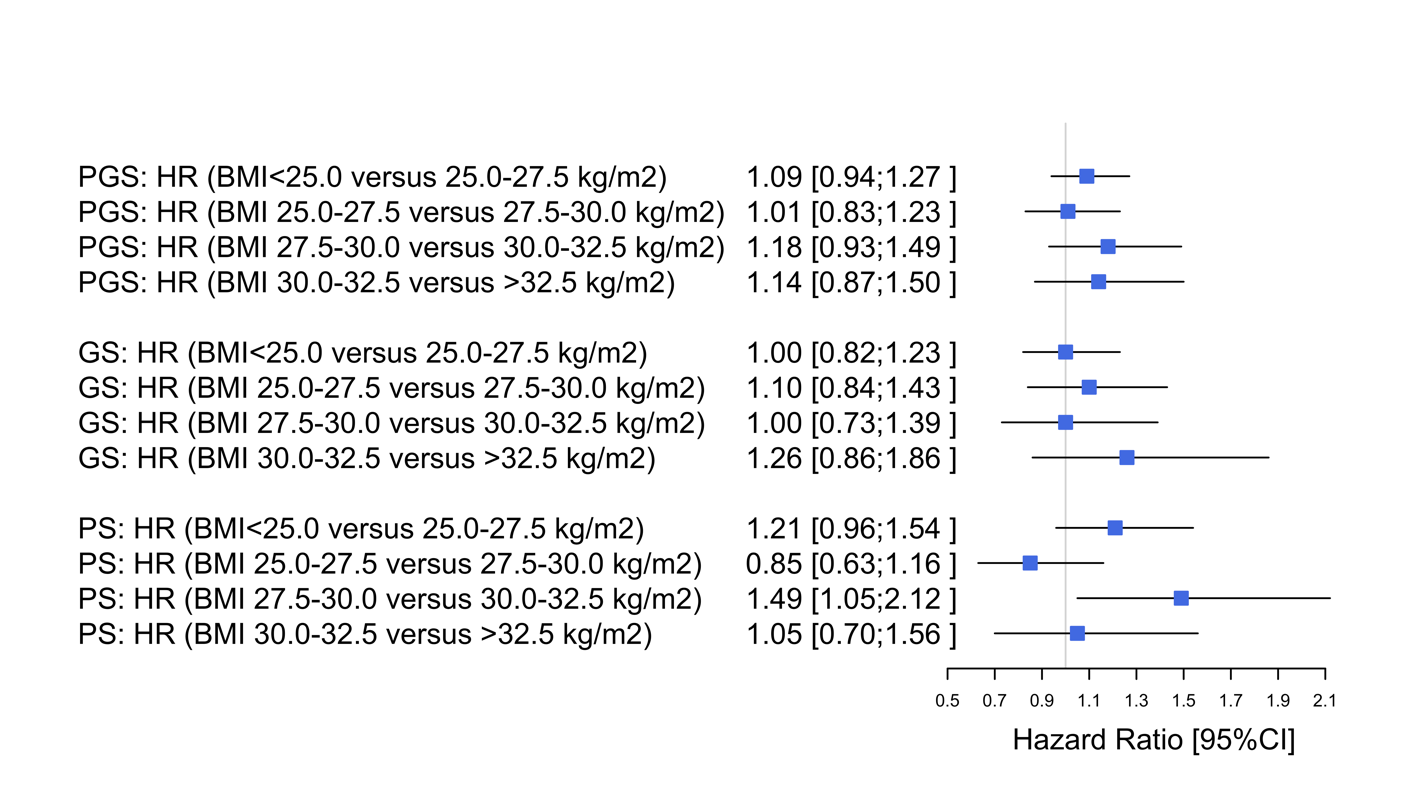


**FIGURE S2. Recipient age-based subgroup analyses to estimate the adjusted hazard ratio (HR) of obese versus non-obese patients for the long-term outcomes: the patient and graft survival (PGS), the graft survival (GS), and the patient survival (PS).**


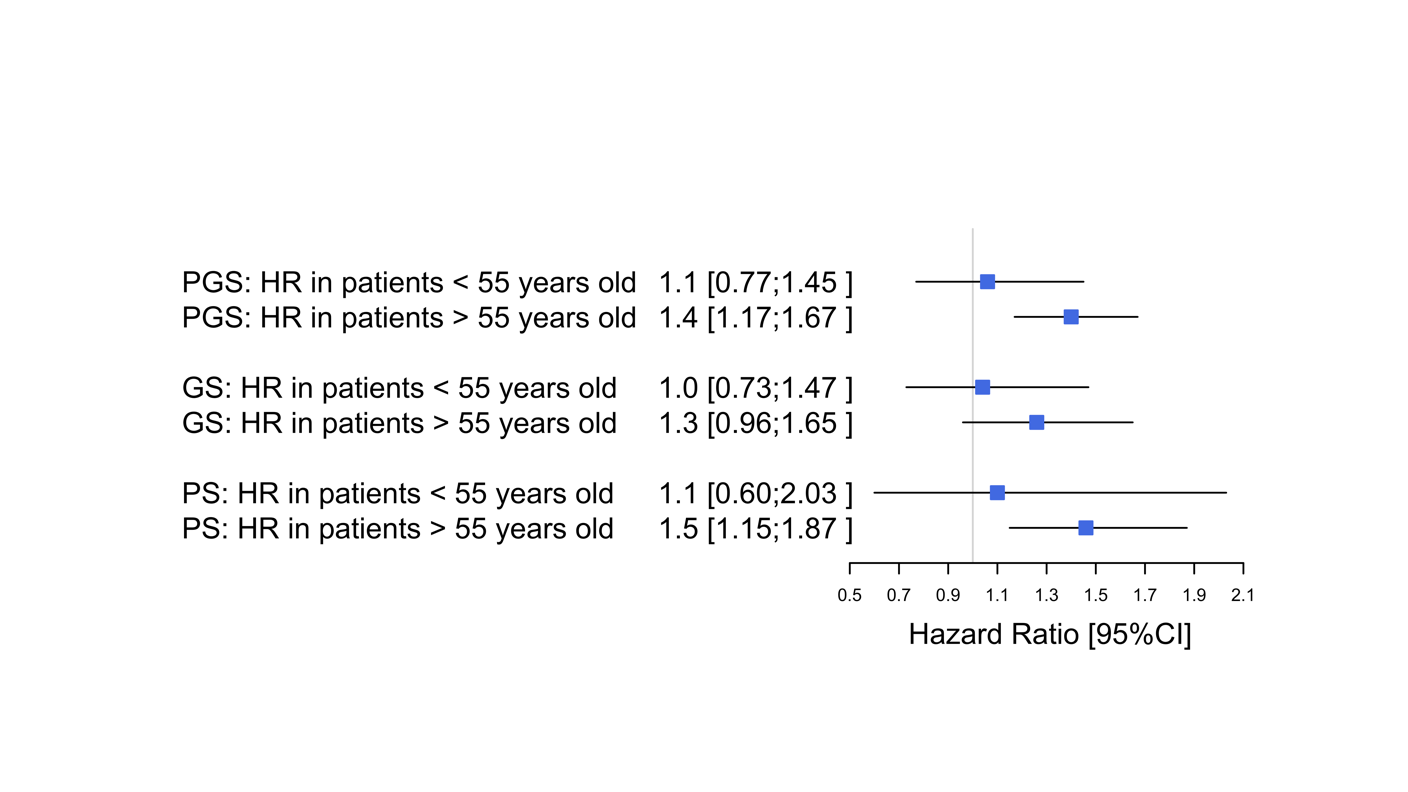

Supplement: Supplementary file 1 — Additional file 1. [file 12882_2021_2278_MOESM1_ESM.docx]
